# Editorial: Therapeutic targeting of splicing variants in cancer

**DOI:** 10.3389/fphar.2023.1206342

**Published:** 2023-05-05

**Authors:** Pouya Sarvari, Karla Rubio, Stephanie Dobersch, Aglaia Ntokou, Verónica Vallejo-Ruiz

**Affiliations:** ^1^ Independent Researcher, Puebla, Mexico; ^2^ International Laboratory EPIGEN, Consejo de Ciencia y Tecnología del Estado de Puebla (CONCYTEP), Instituto de Ciencias, Benemérita Universidad Autónoma de Puebla (BUAP), Puebla, Mexico; ^3^ Laboratoire IMoPA, Université de Lorraine, CNRS, UMR 7365, Nancy, France; ^4^ Human Biology Division, Fred Hutchinson Cancer Center, Seattle, WA, United States; ^5^ Yale Cardiovascular Research Center, Section of Cardiovascular Medicine, New Haven, CT, United States; ^6^ Yale Department of Genetics, New Haven, CT, United States; ^7^ Centro de Investigación Biomédica de Oriente, Instituto Mexicano del Seguro Social, Puebla, Mexico

**Keywords:** alternative splicing, spliceosome, splicing factors, aberrant splicing, pre-mRNA

The cellular process of removing introns from pre-mRNAs and connecting the remaining exons to produce a mature mRNA is termed mRNA splicing. However, exons from the same pre-mRNA can be joined in different combinations by Alternative Splicing (AS), generating diversified mRNA transcripts which can be translated to produce proteins with distinct functions and structures, and yet arise from a single gene. Thus, AS is an essential regulator of gene expression and proteome diversity. Nearly all multi-exon genes undergo AS executed by the spliceosome, regulated by various RNA-binding proteins (RBPs), and aided by cis-acting elements and trans-acting factors. Aberrations in AS lead to tumorigenesis due to either mutation in splicing-regulatory elements of specific cancer genes, a mutation within canonical RNA splicing sites influencing mRNA maturation, or even alterations in the regulatory splicing machinery. Understanding AS in cancer cells would give us better insights into tumor biology and better therapeutic means to control tumor progression.

In this special Research Topic of *Frontiers in Pharmacology*, Li et al. highlighted the effect of AS events on papillary thyroid cancer (PTC) characterization and provided a more precise molecular subclassification for PTC patients by integrating AS profiling for improving medical therapy. Different PTC subtypes exhibit various clinical manifestations and prognoses. However, therapeutic success has been limited by the heterogeneity of PTC at histological and molecular levels. The authors did an outstanding job in the identification of 8834 AS events from 442 PTC patients from the TCGA SpliceSeq database via high-throughput *in silico* analysis using rigorous filtering criteria. Based on six differentially expressed alternative splicing (DEAS) events in all PTC variants, the authors proposed a new subclassification to reclassify PTC and to dissect those with worse prognosis, higher immune heterogeneity, and higher sensitivity to immune checkpoint inhibitors (anti-PD1 therapy). Finally, based on a large-scale perturbation database analysis, the connectivity map (CMAP) platform, the authors suggested orantinib, tyrphostin-AG-1295, and AG-370 for the potential treatment of PTC patients. These findings raise the intriguing possibility of not only detecting splice variants differentially expressed in PTC as a promising diagnostic marker but also of targeting splicing regulatory proteins and global splicing in PTC subtypes with better therapeutic potential. However, more work is needed to pave a route to clinical translation.

Focusing on multi-RTK inhibitors as antitumor agents, the work of Ren et al. centered on the mechanisms underlying the differential effects of RTK inhibitors, including sorafenib, lenvatinib, and regorafenib, in hepatocellular carcinoma (HCC). KEGG pathway analysis of the downregulated proteins identified the top six signaling pathways: spliceosome, nucleotide excision repair, cell cycle, mRNA surveillance, DNA replication, and P53. Moreover, the authors detected 12 downregulated proteins involved in the spliceosome signaling pathway in response to the application of all three RTK inhibitors on the HCC cell line (Huh-7), such as DDX5, DHX15, and eIF4A3, which are involved in spliceosome assembly, and five splicing factors including WBP11, BUD31, Prp19, SF3A1, and UAP56, which aid the removal of introns from strings of mRNA so that the exons can bind together. In addition, SPF30, Dib1, SART1, and SNRPF are involved in integrating the U4/U6.U5 tri-snRNP into the pre-spliceosome. However, more work is needed to identify those specific splicing variants of tumor suppressor genes that arise from the application of RTK inhibitors using transcriptomics. More importantly, combining omics data, such as proteomics and transcriptomics, and their preclinical validation would elucidate how those core splicing factors related to the expression of tumor-specific splicing variants arise from the application of RTK inhibitors before their valuable clinical use as a targeted therapy in HCC.

In a separate review, Apostolidi and Stamatopoulou discussed aberrant splicing in human cancer based on RNA secondary structure. The authors noted that the complex RNA G-quadruplex structures (rG4s) are key regulators of AS and other specific RNA motifs identified as splicing silencers or enhancers. Moreover, the authors addressed the detection of cancer-related aberrant splicing via various detection tools and novel web-based analysis platforms. Finally, the authors elucidated the potential of RNA-based strategies, such as splice-switching antisense oligonucleotides (ASOs), as a therapeutic approach to target AS.

Singh et al. reviewed the role of circular RNA (circRNA) generated by back-splicing in cancer initiation and progression. The authors addressed the regulatory role of circRNAs in cellular processes such as AS, transcription, and translation via various mechanisms. The authors further proposed the regulatory function of the circRNA-miRNA-transcription factor axis in the progression or suppression of cancer, and the targeting of this axis as a potential therapeutic approach for cancer management. CircRNAs are considered potential biomarkers and therapeutic targets in different types of cancer. However, our understanding of the regulation and function of circRNAs is still limited. Moreover, most of the differentially expressed circRNAs in cancer may not necessarily be cancer drivers. Hence, extensive studies on establishing the functional relevance of circRNAs and their products need to be done.

Despite the many milestones achieved in cancer treatment, the role of alternative mRNA splicing has only recently gained attention as a major contributor to cancer initiation and progression. Moreover, AS is emerging as a new source of highly specific biomarkers to classify tumors into different grades, and much remains to be addressed in the future concerning the sheer complexity of the splicing network, including cis-elements, spliceosome assembly, RNA structure, chromatin epigenetic signature influence in AS, and a plethora of trans-elements with antagonistic functions. Taken together, these articles highlight some key areas of interest in this field, from discussing emerging RNA-based therapies to modulate pathogenically spliced isoforms in cancer treatment, such as splice-switching antisense oligonucleotides (ASO) and CRISPR/Cas-based editing, to the identification of splicing variants and splicing factors via the application of high-throughput *in silico* analysis for subclassifying aggressive PTC, to preclinical mechanistic explorations of RTK inhibitors and identification of the downregulated splicing factors, suggesting an alteration in AS, contribute to the observed anti-tumor effects of RTK inhibitors *in vitro*. However, their exact mechanisms driving anti-tumor effects in HCC are still unclear and require further investigation in the setting of the complex AS gene-regulatory network.

